# Point blank: an endoscopic retrieval of an extraluminal bullet

**DOI:** 10.1016/j.vgie.2022.07.003

**Published:** 2022-09-17

**Authors:** Krishna C. Gurram, Sindhura Kolli, George Agriantonis, Renee Spiegel, Josh Aron

**Affiliations:** 1 Department of Gastroenterology and Hepatology, Icahn School of Medicine, Mount Sinai/Elmhurst Hospital, New York, New York; 2 Department of Medicine, New York University Grossman School of Medicine, New York, New York; 3 Department of Gastroenterology and Hepatology, Icahn School of Medicine, Mount Sinai/Elmhurst Hospital, New York, New York

## Abstract

Video 1 Extraluminal bullet retrieval.

Extraluminal bullet retrieval.

A 36-year-old man with no medical history presented with multiple gunshot wounds to the right neck, left axilla, and pelvis. An entry wound in the right buttocks was noted without a corresponding exit wound. A CT scan identified the bullet near the rectum, and a leak from an administered barium enema further demonstrated the location. (Figs. 1 and 2) A laparoscopic diverting colostomy was performed, and advanced endoscopy was consulted for retrieval of the bullet for ballistics and closure of the subsequent rectal defect.

**Figure 1:**
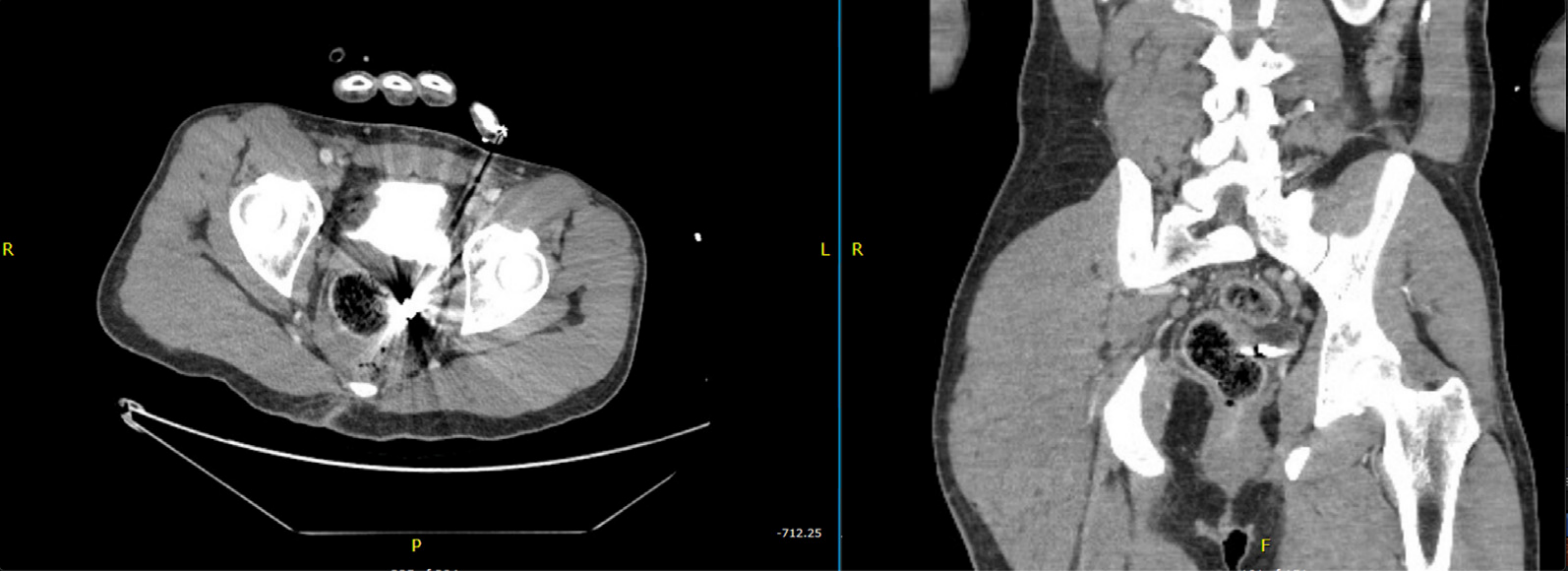
CT scan with bullet.

**Figure 2:**
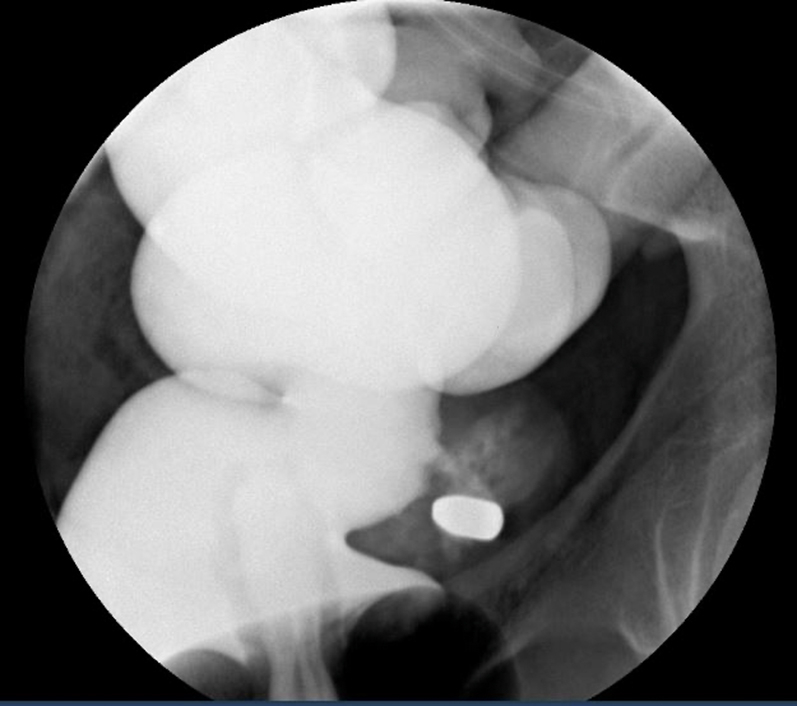
Barium enema with leak.

A gentamicin wash was initiated prior to the introduction of an EGD with a cap. Both the perforated site and distance of the bullet from the rectal lumen were identified, the latter by contrast injected with a stone extraction balloon scope (Figs. 3 and 4). A wire was guided through the balloon into the perirectal space. A sequential balloon dilation up to 10 mm was undertaken to transform the perforated site into a therapeutic window. Unfortunately, the bullet could not be visualized. A fluoroscopic image also confirmed that the scope was not immediately adjacent to the bullet. This approach was abandoned for a more en face window.

**Figure 3:**
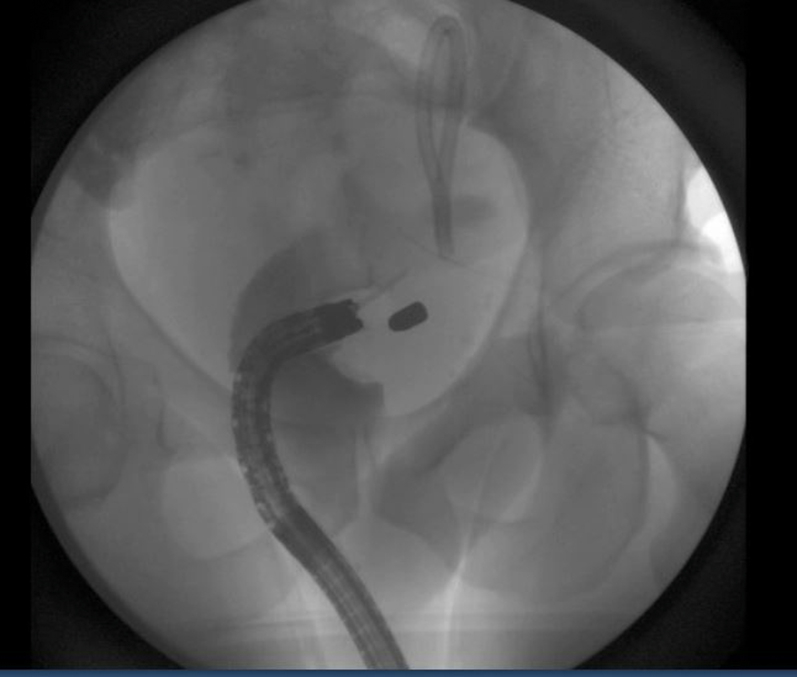
Bullet in relation to scope and lumen.

**Figure 4:**
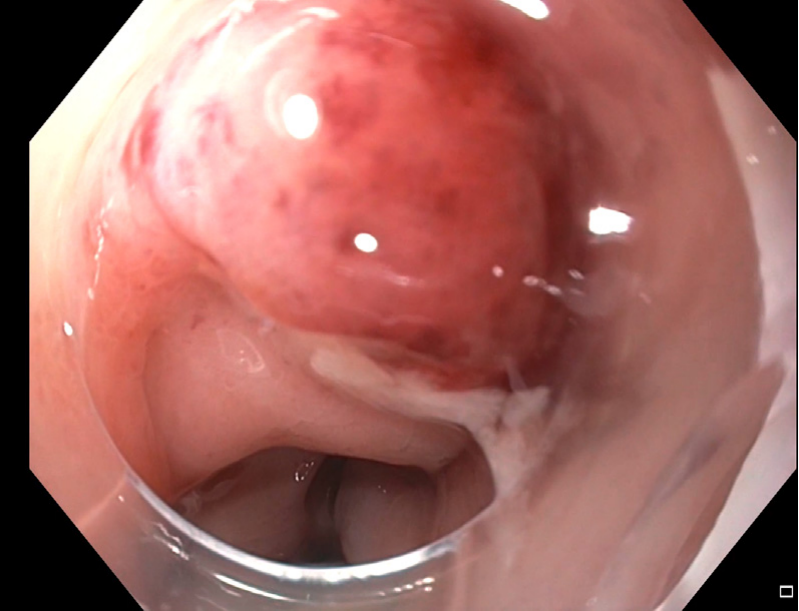
Bullet perforation site.

A linear EUS revealed a hyperechoic bullet outside of the lumen (Fig. 5). A 19-gauge FNA needle was used to puncture the rectal wall to penetrate the perirectal area and abut the bullet. This en face position was confirmed fluoroscopically (Fig. 6). A 0.025 guidewire was introduced into the track and dilatation of this track was commenced with a 3.9 sphincterotome. Further dilation from 4 mm to 10 mm was attained by sequential balloon dilation (Fig. 7). However, the bullet was not within the view.

**Figure 5:**
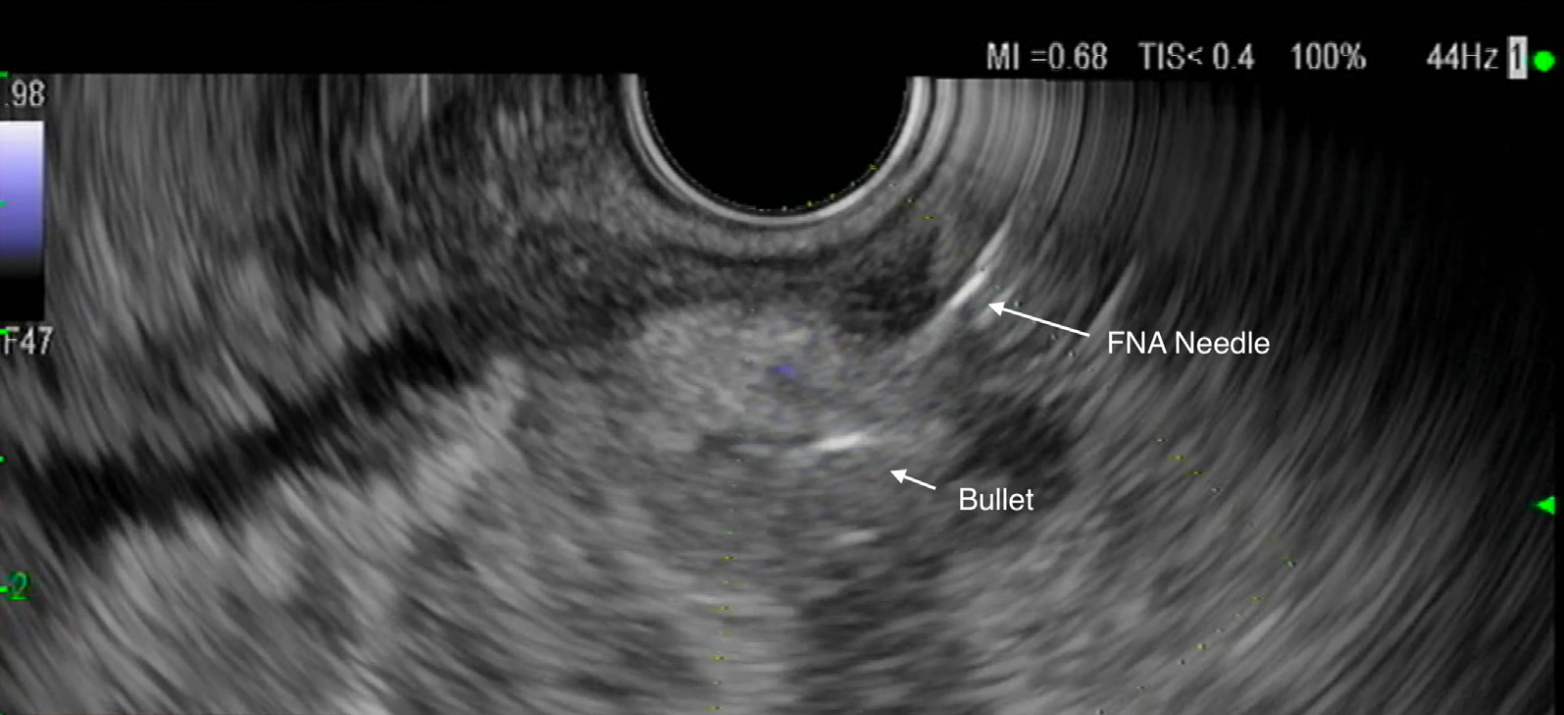
Bullet and FNA needle.

**Figure 6:**
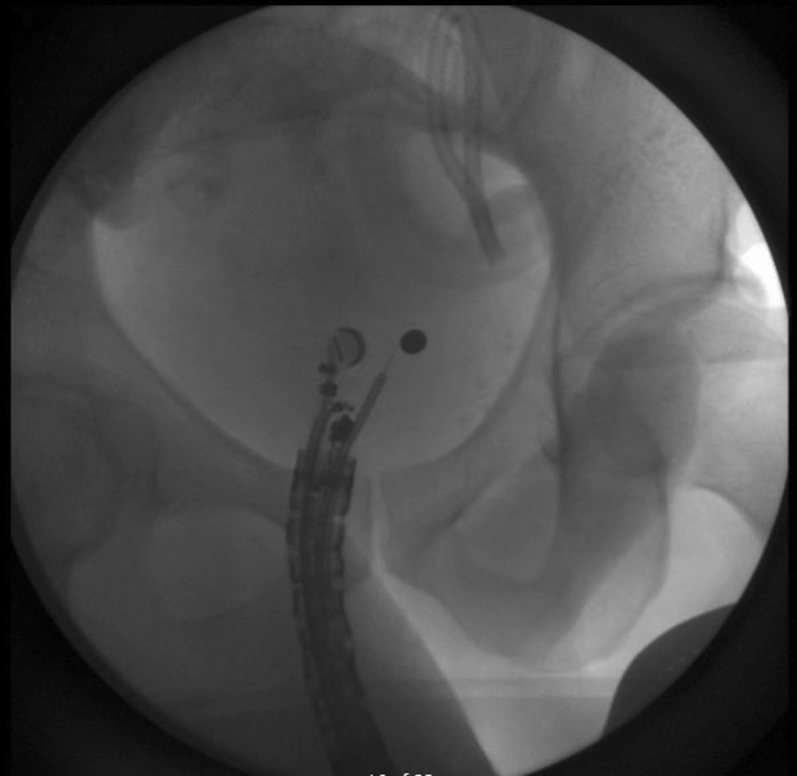
EUS-guided en face needle fluoroscopy.

**Figure 7:**
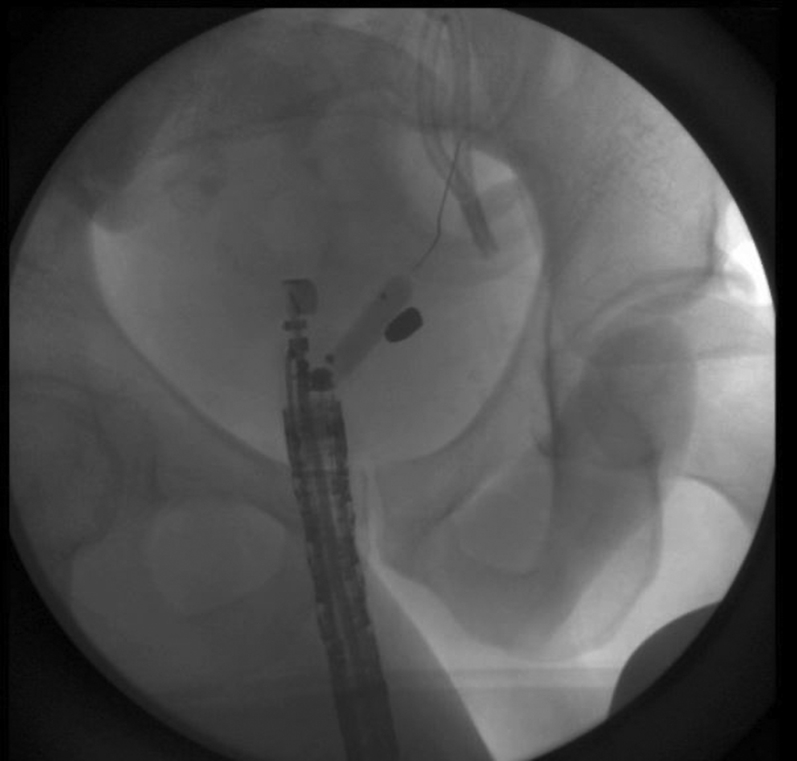
EUS balloon dilation.

A decision was made to further widen the therapeutic window by dissecting the rectal wall layers individually with an insulated-tip knife-2 (Fig. 8). This must be done cautiously and slowly given the high likelihood of coming into contact with the extensive vasculature at this site of the colon and multiple other extraluminal structures surrounding the rectum. This technique finally afforded direct visualization of the bullet (Fig. 9). Despite visualization, the narrowness of the rectal area was another hurdle. A Roth net was originally used, but it was unable to expand in the cramped space nor was it fluoroscopically visualized. Similar limitations were encountered using a basket method. Finally, a retrieval forceps was visualized fluoroscopically and manipulated enough to grasp the bullet and extract the bullet whole (Fig. 10).

**Figure 8:**
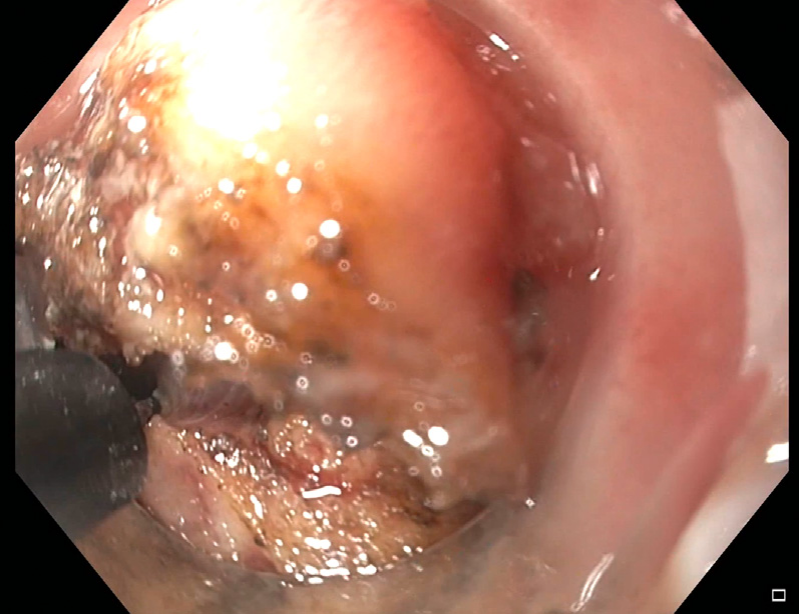
Full-thickness resection.

**Figure 9:**
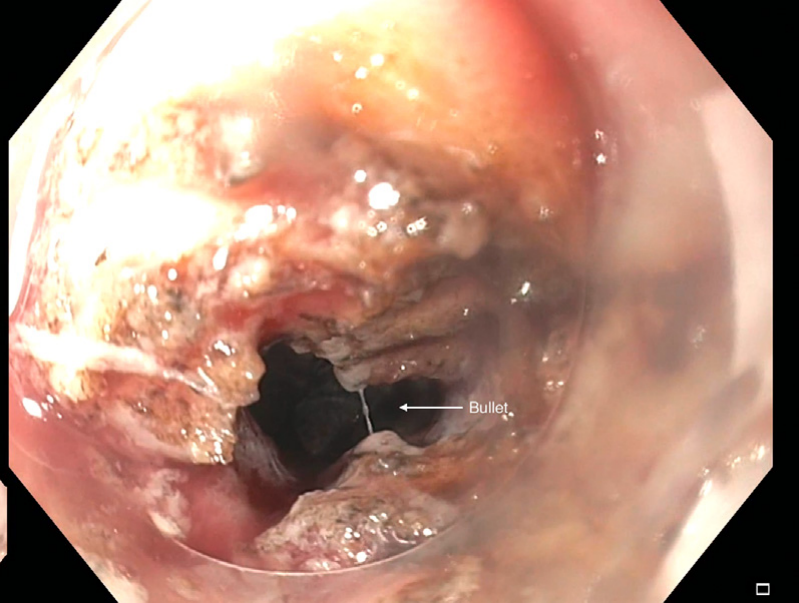
Bullet visualized through opening.

**Figure 10:**
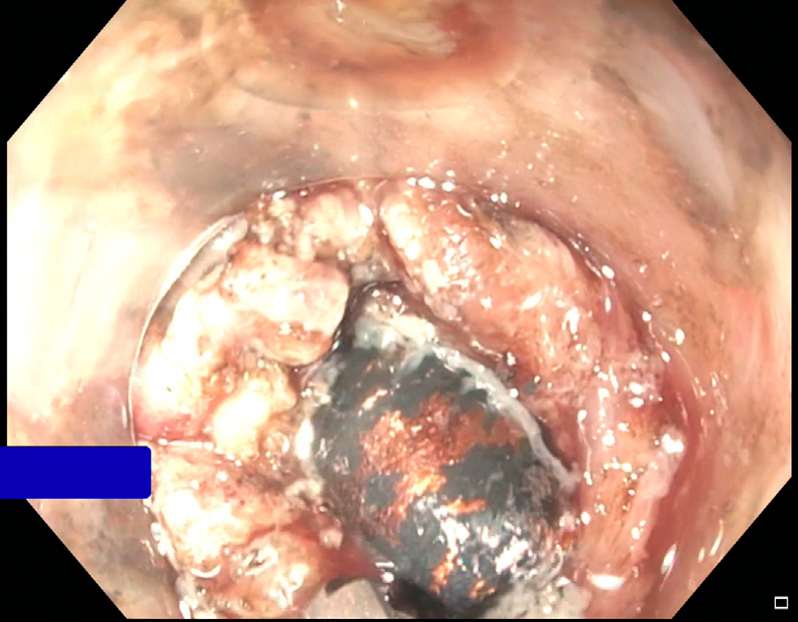
Bullet retrieval with forceps.

The second part of the procedure is to close the large defect that was created. The distal end of the cap was used to estimate the size of the gaping aperture. Since the cap was able to cover the whole lesion a over-the-scope clip was utilized. A twin grasper approximated the two ends of the opening and was pulled into the over-the-scope clip cap. An over-the-scope clip was used successfully for both the opening including the original perforated site. Confirmation was obtained fluoroscopically by injecting contrast through a stone extraction balloon into the lumen with no leakage (Fig. 11). The bullet was noted to be whole without any shards or fragments remaining in the body.

**Figure 11:**
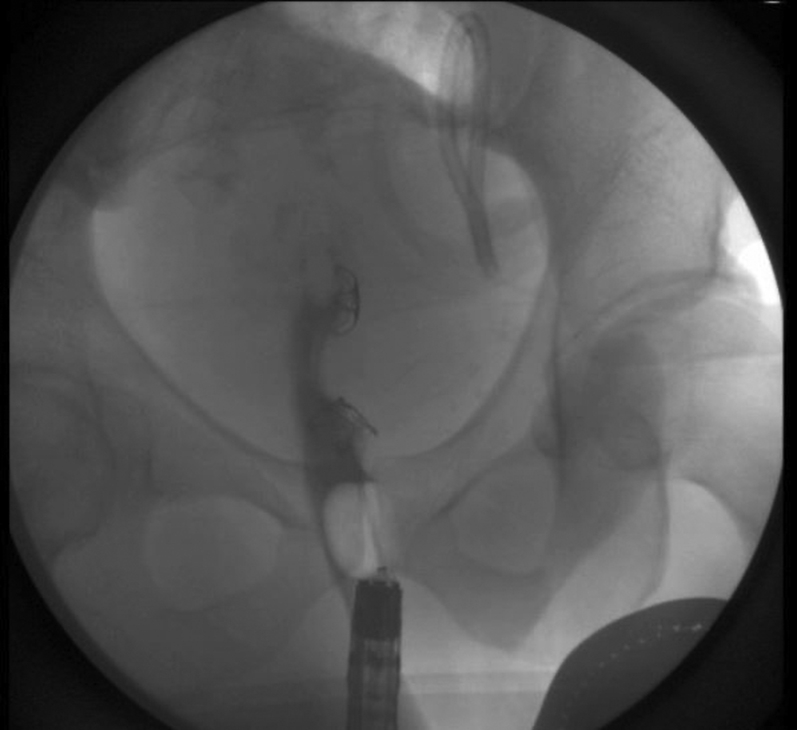
Contrast injection after closure of defects.

This en face method allowed for safe retrieval of the bullet, especially since the colon is highly vascular, is narrow, and despite creating a larger window, allowing for a clean closure. It is important to understand that if one method is not working properly, then reevaluating and changing the technique may be necessary to reach the goal (Video 1, available online at www.giejournal.org).

## Disclosure

*All authors disclosed no financial relationships.*

## Supplementary data

Video 1 Extraluminal bullet retrieval.

